# Leveraging iPSC technology to assess neuro-immune interactions in neurological and psychiatric disorders

**DOI:** 10.3389/fpsyt.2023.1291115

**Published:** 2023-11-09

**Authors:** Christina Michalski, Zhexing Wen

**Affiliations:** ^1^Department of Psychiatry and Behavioral Sciences, Emory University School of Medicine, Atlanta, GA, United States; ^2^Department of Cell Biology, Emory University School of Medicine, Atlanta, GA, United States; ^3^Department of Neurology, Emory University School of Medicine, Atlanta, GA, United States

**Keywords:** induced pluripotent stem cells, neuroimmunity, microglia, neurodevelopment, psychiatric disorders, neurodegenerative disorders

## Abstract

Communication between the immune and the nervous system is essential for human brain development and homeostasis. Disruption of this intricately regulated crosstalk can lead to neurodevelopmental, psychiatric, or neurodegenerative disorders. While animal models have been essential in characterizing the role of neuroimmunity in development and disease, they come with inherent limitations due to species specific differences, particularly with regard to microglia, the major subset of brain resident immune cells. The advent of induced pluripotent stem cell (iPSC) technology now allows the development of clinically relevant models of the central nervous system that adequately reflect human genetic architecture. This article will review recent publications that have leveraged iPSC technology to assess neuro-immune interactions. First, we will discuss the role of environmental stressors such as neurotropic viruses or pro-inflammatory cytokines on neuronal and glial function. Next, we will review how iPSC models can be used to study genetic risk factors in neurological and psychiatric disorders. Lastly, we will evaluate current challenges and future potential for iPSC models in the field of neuroimmunity.

## 1. Introduction

Historically, the brain was considered a site of immune privilege, with minimal immune cell activation and infiltration except during states of infection and disease. However, thanks to technological advances, it is now appreciated that continuous neuro-immune crosstalk is essential in maintaining brain homeostasis (1–3). Microglia, the brain resident immune cells perpetually survey the central nervous system (CNS), not only in defense against invading pathogens but also to phagocytose dying or malfunctioning cells as well as protein aggregates, to provide trophic support to neurons, and to shape neuronal activity (1, 4). The importance of neuro-immune interactions is underscored by the emergence of disease when this crosstalk is disrupted. Indeed, an increasing number of genetic risk factors for neurodegenerative disorders such as Alzheimer’s disease (AD) are found to be located in genes essential for microglia function (5). Additionally, patients with psychiatric disorders such as major depressive disorder (MDD), schizophrenia (SCZ), or bipolar disorder (BD) often exhibit abnormally high levels of systemic inflammation and microglial activation compared to the general population (6, 7). Interestingly, elevation in circulating inflammatory markers can precede the symptom onset, for example the first episode of psychosis in SCZ, suggesting a mechanistic role for inflammation in disease etiology (8). Lastly, postmortem brain tissue from patients with psychiatric or neurodegenerative disorders often show signs of microglial and astrocyte activation, representative of an inflammatory state in the CNS (9, 10). Despite considerable advances in therapeutic care for neuropsychiatric disorders, disease mechanisms often remain incompletely understood, hampering the development of novel, curative clinical interventions.

Neuropsychiatric disorders are commonly attributed to the interplay of genetic predisposition and environmental exposure. This complex interaction is difficult to model *in vitro* and animal studies as they do not adequately reflect the genetic architecture and heterogeneity found in humans. Additionally, significant species-specific differences exist with regard to the brain. Core microglia functions are conserved across evolution, but human microglia exhibit distinct transcriptional and functional programs, particularly with regard to disease associated genes (11). The advent of human embryonic or induced pluripotent stem cell (ESC and iPSC, respectively) technology has revolutionized the field of neuro-immunology. Indeed, generation of various human neuronal and glial subtypes allows the study of neuro-immune interactions in clinically relevant models. In this article, we will briefly summarize current stem cell-based models of the CNS before reviewing studies assessing the role of environmental or genetic factors in neurological and psychiatric disorders. Finally, we will discuss limitations of iPSC models and highlight potential for future studies.

## 2. Stem cell-based models of the human central nervous system

**Figure 1:**
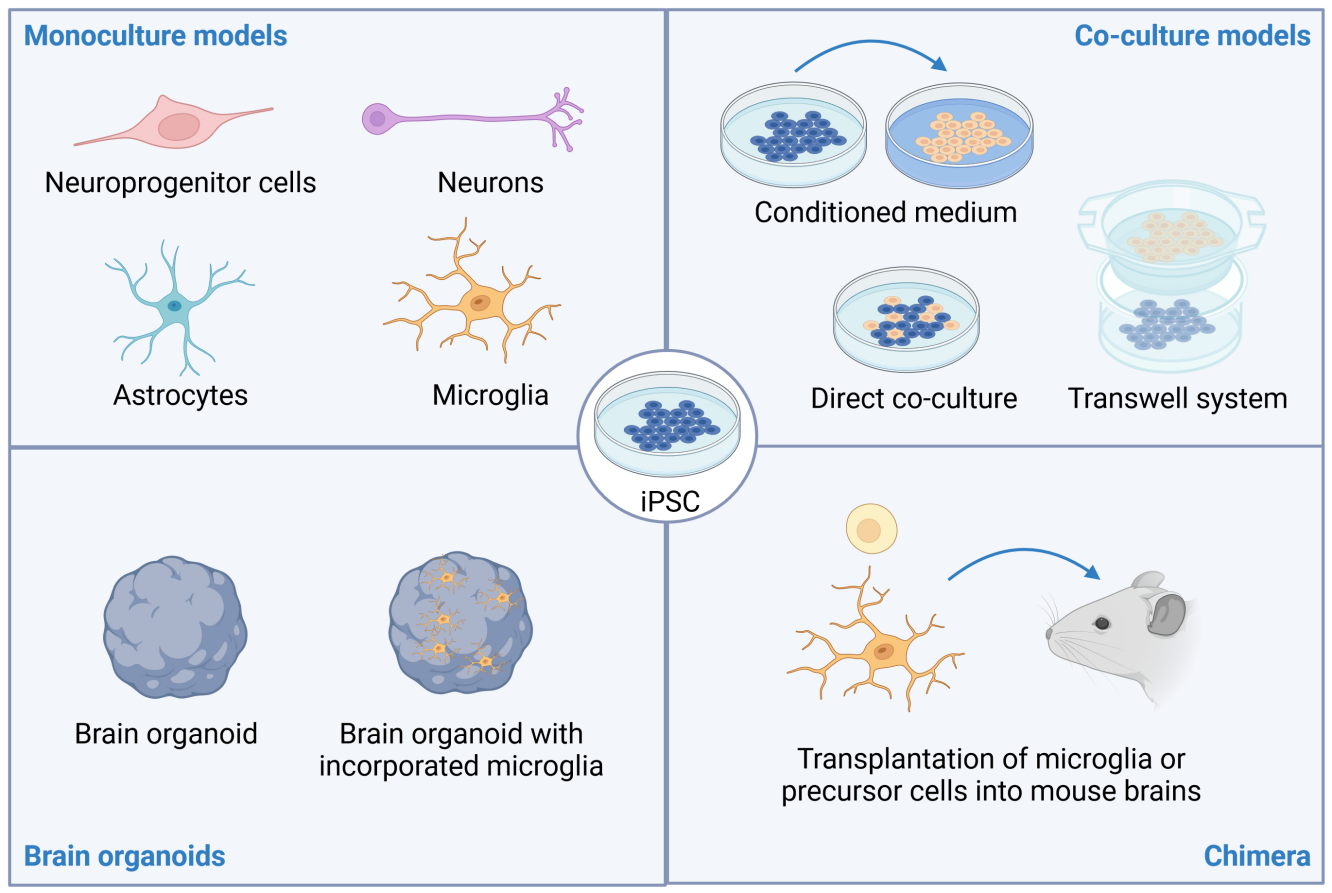
Stem cell models of neuro-immune interactions. Induced pluripotent stem cells (iPSC) can be differentiated into various types of brain resident cells including neuroprogenitor cells, neurons, astrocytes, and microglia. To study neuro-immune interactions, co-culture models have proven insightful. These can include adding conditioned medium from one cell type (e.g., iPSC-derived microglia) to cultures of another cell type (e.g., iPSC-derived neurons), direct co-culture of two or more cell types, or co-cultures using microfluidic or transwell systems to limit direct cell-to-cell contact. Brain organoids, with or without incorporation of microglia can also be used to study neuropsychiatric disorders. Lastly, transplantation of microglia or microglia precursor cells into rodent brains promotes maturation of the microglia and allows studying neuro-immune interactions in an *in vivo* setting. This image was created using Biorender.

Over the past decade, a growing number of iPSC-based models of neuro-immune interactions have been published (Figure 1). It is beyond the scope of this article to summarize all available protocols, but the reader is referred to several excellent reviews comparing advantages and limitations for various protocols of iPSC-derived neurons (12, 13), microglia (14–16), and astrocytes (12, 17).

### 2.1. Neurons and brain organoids

Differentiation of iPSC into cortical neurons is typically achieved by forced expression of the transcription factor neurogenin-2 (NGN2) and exposure to growth factors and other molecules known to promote neurodevelopment (18). More recent protocols similarly allow development of other neuronal subtypes such as GABAergic neurons or glial cells like astrocytes or oligodendrocytes through transcription factor overexpression (19–21). Other protocols rely on the timed exposure to growth factors, hormones, and cytokines to differentiate iPSC into specific subsets of brain cells (22). While the latter approach may resemble embryonic development more accurately, the former approach allows faster and more consistent generation of cells (13). In addition to monocultures of various cell types, ESC or iPSC can also be differentiated into so-called brain organoids or spheroids—self-organizing 3D structures that recapitulate human embryonic brain development. These (often brain region specific) organoids have initiated a new era in studying molecular cascades in the human brain and hold tremendous potential for further discoveries on human brain function in health and disease (23–25). In the initial stages, brain organoids largely consist of neural progenitor cells with mature neurons and astrocytes subsequently develop at later stages. Brain organoids functionally mature as evidenced by emergence of electrophysiological properties (26). However, brain organoids typically lack microglia due to their distinct embryonic origin—neuronal cell types are derived from the ectoderm while microglia derive from the mesoderm.

### 2.2. Astrocytes

Various protocols exist to generate astrocytes from iPSC. Often, a multistep process is applied; differentiating iPSC into neural progenitor cells (NPCs) followed by lineage commitment to astrocyte precursors that gradually mature into astrocytes (12, 17, 27). Since these protocols can be time consuming, several groups have developed protocols to initiate immediate differentiation of iPSC into astrocytes. These protocols commonly rely on overexpression of lineage-driving transcription factors such as NFIA/B or SOX9 (19, 28). Validation of astrocyte identity and functionality include assessment of astrocyte markers AQP4, S100B, or GFAP, response to inflammatory stimuli, glutamate uptake, and support of neuronal functions, for example evidenced through neuronal network maturation in co-culture models (19, 29–31). Generally, iPSC-derived astrocytes *in vitro* resemble fetal human astrocytes more closely than adult human astrocytes (29, 30).

### 2.3. Microglia

Similar to astrocytes, iPSC-derived microglia (iMG) are commonly generated in a two-step manner by first initiating differentiation toward the mesoderm, generating erythromyeloid progenitor or hematopoietic progenitor cells before polarizing cells to a microglial state (14, 16, 32–34). Recent publications also demonstrate that microglia can be generated through overexpression of transcription factors such as CEBPB and/or PU.1. These protocols significantly reduce the time required for microglia development and may facilitate modification by molecular tools such as CRISPR (35, 36). To validate functionality, iMG are often stimulated with a combination of lipopolysaccharide (LPS) and interferon (IFN)-γ which leads to secretion of pro-inflammatory cytokines (34, 36). It should be noted that LPS is a bacterial cell wall component and hence not necessarily a physiological stimulus. However, due to its well-defined downstream responses in monocytes, macrophages, and microglia, it remains commonly used. Other inflammatory stimuli such as IFN-γ or IL-1β lead to distinct, yet similar responses (32). Further hallmarks of functional microglia include phagocytosis of beads or other particles, migration to sites of injury and ADP/ATP evoked changes in calcium transients (15, 32, 34, 37). Sometimes, microglia are labeled as M1 or M2 microglia, mirroring the nomenclature historically used for inflammatory (M1; induced by IFN-γ stimulation) and anti-inflammatory (M2; induced by treatment with IL-4) macrophages. However, this naming system has received criticism as myeloid cell polarization occurs on a spectrum and *in vivo* phenotypes do not neatly correspond to an M1 or M2 state. Therefore, it is now generally recommended to avoid these terms (38, 39). Technological advances in (single cell) RNA sequencing have allowed the characterization of microglial states found in physiological and pathological conditions. Transcriptomic signatures that are shared across disease-states and species have been developed. One example includes the disease associated microglia (DAM) phenotype, characterized by altered lysosomal and lipid metabolism. Microglia exhibiting DAM properties are enriched in postmortem brain tissue of patients with neurodegenerative disorders as well as their corresponding animal models (40, 41). Recent efforts have demonstrated that *in vitro* treatment of iMG with disease relevant stimuli (e.g., myelin debris, apoptotic neurons, or synthetic amyloid beta) can induce transcriptomic states comparable to those found *in vivo*, including the DAM signature (42). It should be noted that the DAM signature, while widely used, is still controversial in the microglia research community (39).

### 2.4. Co-culture models

Interestingly, studies consistently demonstrate that iMG undergo significant functional maturation and resemble their *in vivo* counterpart more closely when they are co-cultured with neurons, integrated into brain organoids or transplanted into mouse brains (34, 43–45). Conversely, when iMG are added to brain organoids, neuronal maturation is accelerated, exemplified by synapse pruning and acquisition of more mature electrophysiological properties (44, 46). Together, this highlights the importance of neuro-immune crosstalk for brain function. Transplantation of iMG or hematopoietic precursor cells into mouse brains to generate chimera is an especially useful model to study human microglial behavior in an *in vivo* model and holds great promise to identify novel therapeutic targets for many neuropsychiatric diseases. For example, transplanted human microglia are capable of phagocytosing myelin debris in the commonly used cuprizone model of multiple sclerosis (47), exhibit DAM signatures and migrate toward amyloid plaques in the 5XFAD model of Alzheimer’s Disease (43).

## 3. Modeling the impact of environmental stressors

**Figure 2:**
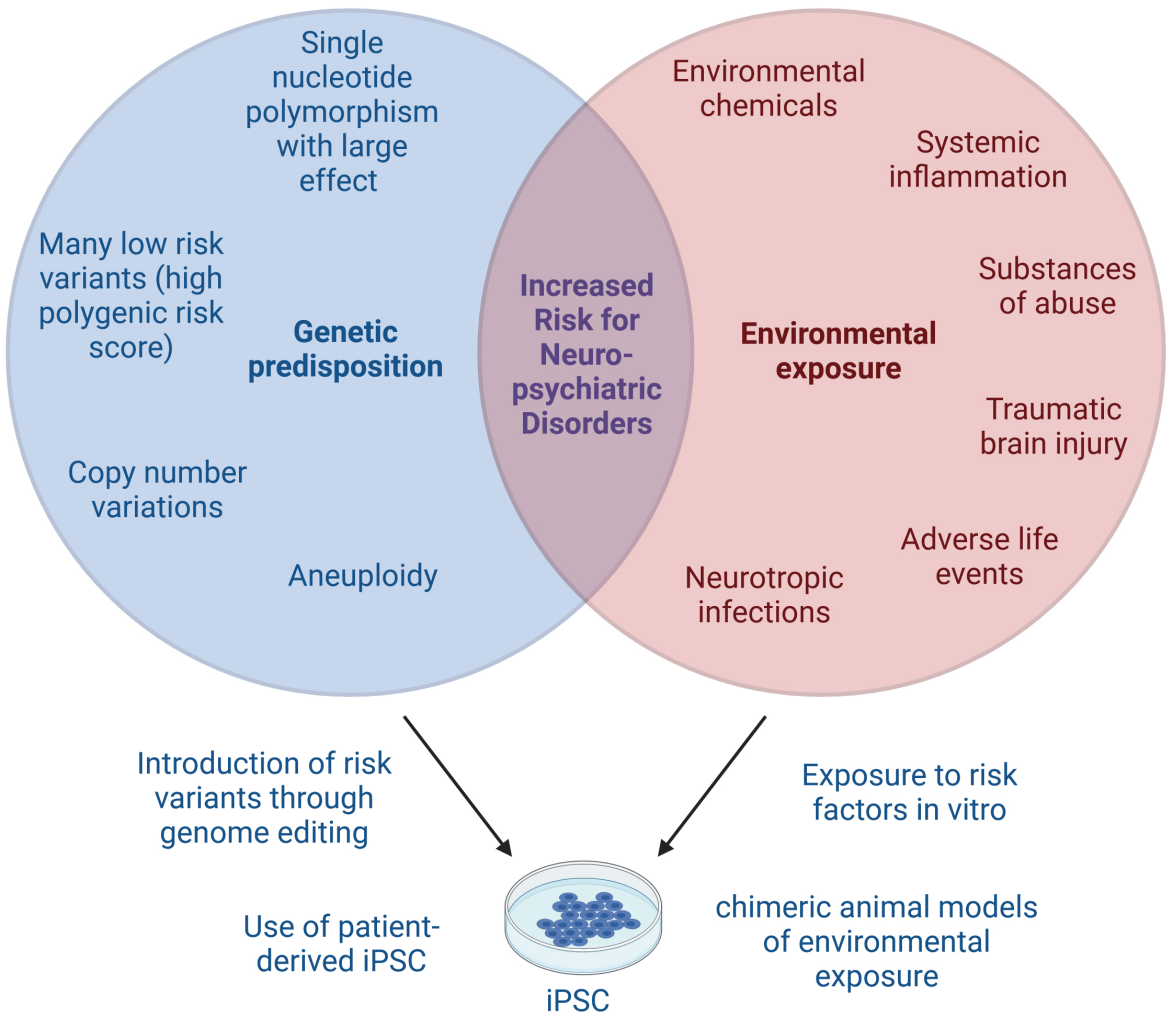
Gene × environment interactions in neuropsychiatric disorders. Many neuropsychiatric disorders arise from complex interplay of genetic predisposition and environmental exposure. Genetic risk factors include single nucleotide polymorphisms with small or large effect size that together may result in a high polygenic risk score. Copy number variations or aneuploidy can also increase the risk for neuropsychiatric disorders. Known environmental risk factors include certain chemicals, systemic inflammation, substances of abuse, traumatic brain injuries, adverse life events and neurotropic infections, among others. iPSC models can be used to study genetic risk factors, either by using patient-derived iPSC or by introducing known genetic risk variants, for example using CRISPR. Environmental risk factors can be studied by addition of risk substances to *in vitro* cultures or by transplantation of iPSC into animal models of neuropsychiatric disorders. Lastly, the interaction of genetic and environmental risk factors can be modeled using iPSC by combination of the above described techniques. This image was created using Biorender.

In most cases, neuropsychiatric disorders are thought to arise through interplay of environmental and genetic risk factors (Figure 2) (48). Environmental stressors commonly associated with psychiatric or neurodegenerative disorders include early life adverse events, psychosocial stress, chronic low-grade inflammation, viral infections, and exposure to certain chemicals (48, 49). In this section, we will highlight iPSC-based studies that have assessed the impact of neurotropic viruses, inflammatory cytokines, or substances of abuse on neuroimmune function. For a summary, please refer to Table 1.

**Table 1:** Stem cell models evaluating environmental stressors in neuro-immunity.

Environmental stressor	Cell type	Effect of stressor in iPSC model	References
Neurotropic viruses			
Zika virus	NPCs	Upregulation of immune response pathways including interferon and inflammatory responses	(50)
	Cranial neural crest cells	Production of LIF, IL-6, and VEGF; only low levels of interferons produced	(51)
	Brain organoids ± microglia	Upregulation of immune response pathways including interferon and antiviral responses; microglia become ameboid; and increased synaptic pruning	(52, 53)
La Crosse virus	Brain organoids	Upregulation of interferon response genes; treatment with type I interferons reduced viral-induced cell death	(54)
Japanese encephalitis virus	Brain organoids	Upregulation of interferon response genes and production of IFN-β	(55)
Toxoplasma gondii	Brain organoids	Upregulation of type I interferon responses	(56)
Herpes Simplex Virus	Brain organoids	Upregulation of inflammatory genes	(57)
	Choroid plexus organoids with incorporated microglia	HSV leads to upregulation of the cGAS-STING pathway in the microglia, resulting in secretion of IFN-α. This limits viral load and HSV-induced barrier disruption.	(58)
Cytomegalovirus	Brain organoids	Upregulation of immune response genes	(59)
SARS-CoV-2	Microglia, brain organoids	Upregulation of antiviral and inflammatory responses, mainly by microglia and astrocytes.	(60–63)
Human immunodeficiency virus	Microglia	More susceptible to macrophage-tropic than T cell tropic HIV; sustain viral replication; and production of pro-inflammatory cytokines	(64–66)
	Brain organoids + microglia (cell line)	Pro-inflammatory cytokine production; apoptosis; reduced synapse density	(67)
Cytokines			
IFN-γ	NPCs	Upregulation of anti-viral, antigen presentation and innate immune response genes; increased neurite length and number	(68)
	Astrocytes	Increased secretion of complement component C4	(69)
IL-6	Neurospheres	Apoptosis; premature neuron formation	(51)
	Neurons	Gliogenesis; JAK–STAT activtion	(70)
	Brain organoids	Gliogenesis; upregulation of immune response genes, particularly in radial glia	(71)
LIF	Neurospheres	Apoptosis; premature neuron formation	(51)
IL-1α, TNF-α, and C1q	Astrocytes	Upregulation of immune response genes; secretion of pro-inflammatory cytokines; reduced phagocytosis; reduced glutamate uptake; impaired support of neuronal function; increased neurotoxicity	(29, 31, 72)
		Similar phenotypes also observed with other combinations of pro-inflammatory cytokines	(73–75)
Substance use disorder			
Oxycodone	Brain organoids	Upregulation of type I interferon response genes, particularly in choroid plexus cells	(76)
Methamphetamine	Brain organoids	Upregulation of immune response genes; production of IL-6; astrogliosis; and NLRP1 expression in astrocytes	(77)
Toxicants			
Cadmium	Brain organoids	Upregulation of JAK–STAT signaling; IL-6 production; astrocyte activation	(78)

### 3.1. Infections of the CNS

Neurotropic pathogens are a major cause of disease burden yet remain difficult to study as animal models are often inadequate due to host-specific tropism. In recent years, iPSC models have bridged this gap and yielded important insight into disease mechanisms. In the following paragraphs, we will highlight a few iPSC based studies of neuro-immune crosstalk evoked by neurotropic viruses. The reader is referred to current reviews on stem cell models of neurotropic infections for a more detailed discussion of the virology and neuronal responses (79, 80).

Perhaps one of the most studied neurotropic pathogens using iPSC-based models is Zika virus (ZIKV), a mosquito-borne flavivirus that, when transmitted to humans, can cause Guillain–Barré syndrome in adults and microcephaly in the developing human fetus (81). iPSC models were essential in demonstrating that human NPCs are particularly susceptible to ZIKV infection and in identifying potential therapeutic targets (82–84). Infection of NPCs or brain organoids leads to immediate upregulation of immune response pathways, particularly interferon and antiviral responses resulting in astrogliosis (50, 52, 53, 85). At later time points post infection, apoptosis and downregulation of transcripts related to nervous system development is observed, resulting in smaller sized organoids (51, 53, 86). Interestingly, quality and strength of the interferon response varies between different strains of ZIKV and is also distinct of the transcriptomic response induced by DENV despite many commonly affected pathways (50). Inhibiting inflammation or promoting interferon responses can partially rescue the detrimental effects imparted by ZIKV (52, 53, 86). Production of interferons may not only be beneficial for neuronal function but also be important for maintaining BBB integrity. Indeed, models of iPSC-derived human brain microvascular endothelial cells to study virus-BBB interactions have revealed that an inflammatory stimulus like LPS or TNF-α reduces barrier integrity and facilitates monocyte migration through the BBB while treatment with IFN-λ has the opposite effect (87).

Induction of interferon responses and its potentially beneficial effects have also been demonstrated in iPSC-models for other neurotropic pathogens including La Crosse virus (54), Japanese Encephalitis Virus (55), Toxoplasma gondii (56), and Herpes Simplex Virus (HSV) (58). For example, HSV infection of iPSC-derived NSCs or neurons resulted in apoptosis and impaired neuronal differentiation, which was also validated in brain organoid models where HSV led to reduced cortical plate thickness and gliosis (57). Interestingly, integration of microglia in a choroid plexus organoid model limited viral load and barrier disruption induced by HSV. This protective effect was conferred through cGAS-STING pathway activation in microglia, resulting in IFN-α secretion (58).

Lastly, iPSC-derived brain cells are also an excellent model to study glia-tropic viruses. For example, accumulating data, including from iPSC models, suggest that SARS-CoV-2 can infect and replicate in astrocytes, and readily elicit antiviral and inflammatory responses in astrocytes and microglia (60–62, 88). Another prominent example of glia specific viral infection is HIV, where replication competent virus likely persists in microglia even in the presence of effective antiretroviral therapy (89). These viral reservoirs in the CNS, together with systemic changes in the microbiome and immune system, likely contribute to HIV associated neurocognitive dysfunction, also known as HAND (90). Stem cell models have replicated *in vivo* findings showing that HIV does not infect iPSC-derived neurons or astrocytes but can readily infect iMG. Infection occurred preferentially with macrophage-tropic strains compared to T cell-tropic strains and induced pro-inflammatory cytokines upon infection (64–66). Incorporation of HIV-infected microglia also led to cell death and reduced synapse density (67). Treatment of HIV-infected iMG with clinically used anti-retrovirals reduced the viral load but only partially rescued the virus-induced inflammation and cytokine production (65), highlighting the potential of iPSC to generate clinically relevant models for HAND. Undoubtedly, future iPSC-based studies will yield further insight into the mechanisms of the neurotropic infections discussed here. They also hold great potential for examining neuro-immune interactions of less studied neurotropic viruses such as Powassan or Nipah virus.

### 3.2. The role of cytokines in shaping neuro-immune interactions

Several of the above summarized studies also examined the direct effects of virus-induced cytokines on neuronal function. Indeed, treatment of neurospheres with LIF or IL-6 (cytokines upregulated after ZIKV infection) led to apoptosis and premature neuron formation which could be a mechanism contributing to microcephaly *in vivo* (51). Clinically, high levels of circulating IL-6 have been associated with psychiatric disorders such as MDD or BD (7). IL-6 is also a hallmark cytokine of maternal immune activation (MIA), an umbrella term to describe the epidemiological link between sterile or infection-induced inflammation during pregnancy with higher rates of neuropsychiatric disorders in the off-spring (91). Modeling MIA *in vitro* is challenging as disease etiology is incompletely understood but treatment of developing brain cells with pro-inflammatory cytokines linked to MIA has yielded valuable mechanistic insight. Indeed, transient exposure to IL-6 during long-term neuronal culture or brain organoid development increases GFAP expression and gliogenesis (71, 70). Radial glia are particularly responsive to IL-6 and upregulate transcripts enriched in protein translation and immune response pathways (71). IFN-γ is another key cytokine implicated in MIA and associated with neurodevelopmental disorders like autism spectrum disorder (ASD). Transient exposure of NPCs to IFN-γ increased neurite length and neurite number (68). Additionally, not only were anti-viral, innate immune response and antigen presentation pathways upregulated, the transcriptomic changes induced by IFN-γ also showed significant enrichment for ASD and SCZ risk genes (68).

It is becoming increasingly apparent, that astrocytes are quickly adapting to changes in their environment and that different astrocyte states can have beneficial or detrimental roles in many neuropsychiatric disorders (92, 93). A landmark study by Liddelow et al. (94) demonstrated that the secretion of pro-inflammatory cytokines by stimulated microglia in turn activates astrocytes, so-called reactive astrocytes that are neurotoxic. *In vitro*, reactive astrocytes can be generated by treating astrocytes with a combination of the cytokines IL-1α, TNF-α, and the complement component C1q (29). Such stimulated astrocytes are often referred to as A1 reactive astrocytes, while A2 reactive astrocytes are considered more neuroprotective, mirroring the nomenclature used for activated myeloid cells (94). However, as mentioned above, binary division of glial cell states does not necessarily correspond to glial phenotypes *in vivo* and may significantly mask the complexity of their malleability and should therefore be avoided (95). Indeed, a recent study performing CRISPR droplet sequencing of iPSC-derived astrocytes treated with IL-1α, TNF-α and C1q identified two distinct transcriptomic clusters of reactive astrocytes which were characterized by IL-1/IL-6 or TNF/IFN driven signaling, respectively (31). While the presence of these two clusters was validated across different models and species using publicly available transcriptomic data, if and how these cell states are functionally divergent remains to be determined. Initial studies examining astrocyte activation were performed using animal models and postmortem brain tissue. Despite significant inter-species differences in astrocyte function (96, 97), iPSC-based models of human astrocytes consistently validate that reactive astrocytes secrete proinflammatory cytokines, display decreased phagocytosis, reduced glutamate uptake, impaired support of neuronal function, and heightened neurotoxicity (29, 31). These phenotypic changes are generally also observed when other pro-inflammatory cytokine cocktails are used for glial activation, although the dynamics and strengths of astrocyte polarization may vary (73–75). Interestingly, astrocyte activation includes upregulation of molecules involved in antigen-presentation (72, 75), supporting that astrocytes may play a crucial role in recruitment and activation of peripheral immune cells (98). iPSC-derived astrocytes have also been instrumental in identifying surface markers for human astrocytes [CD49f as pan-astrocyte marker (29) and VCAM1 as reactive astrocyte marker (31, 72)] which will undoubtedly propel future studies by permitting isolation and further characterization of specific cell subsets. Additionally, surface markers facilitate staining and ensuing quantification of cell populations, for example demonstrating that VCAM1+ astrocytes are enriched in human postmortem brain tissue from patients with hypoxic–ischemic encephalopathy but not AD (31). Lastly, iPSC-based models of astrocyte activation provide a platform to screen compounds with therapeutic potential in neuroinflammation (69, 75).

Despite all the above summarized evidence for gliosis and neurotoxicity induced by cytokines, it is worth noting that homeostatic cytokine signaling is essential for neuro-immune crosstalk and survival of brain cells and modulation of neuronal function (99). Indeed, iPSC-based models of glia cell development rely on the addition of cytokines to achieve successful differentiation into the desired cell types. Increasingly, stem cell models are developed to characterize the homeostatic and beneficial roles of cytokines. For example, using a microfluidic device containing iPSC derived microglia on one side and iPSC derived neurons and astrocytes on the other side, McAlpine et al. elegantly demonstrated that IL-3 secreted by astrocytes induces increased migration of microglia; this IL-3 dependent activation provided benefits in a mouse model of AD through reduced plaque burden (100).

### 3.3. Neuroinflammation in iPSC-based models of substance use disorders

Induced pluripotent stem cell models are also being developed to study molecular effects of substances of abuse on neuronal function. Several substances of abuse induce inflammatory responses in brain organoids supporting a role for neuroinflammation in long-term health outcomes of substance use disorders. For example, treatment of brain organoids with methamphetamine led to upregulation of immune response pathways and cytokine signaling, including production of IL-6 and astrogliosis (77). Similarly, exposure to oxycodone led to induction of type I interferon response pathways in a brain organoid model, particularly in cells annotated as choroid plexus cells (76). Ethanol exposure as a model of alcohol use disorder led to NLRP3 inflammasome activation in NPCs (101). Lastly, treatment of iMG with a synthetic μ-opioid receptor agonist induced pro-inflammatory responses including inflammasome activation, cytokine secretion and morphological remodeling (102). Interestingly, these effects could be partially reversed with a cannabinoid receptor 2 agonist. Given the increasing number of states legalizing cannabis and the well documented association between cannabis consumption and SCZ, further studies will be required to determine mechanisms of the CNS immune response to cannabinoids and other substances of abuse and assess whether neuroinflammation could be a suitable target for treating substance use disorders.

## 4. Modeling the impact of genetic variation on neuroimmunity in neuropsychiatric disorders

While exposure to environmental stressors significantly increases the risk for neuropsychiatric disorders, the individual genetic architecture is important in disease etiology. Genetic architecture describes the sum and complex interaction of all genetic variations, such as single nucleotide polymorphisms and copy number variations that contribute to (disease) phenotypes (103). The heritability for psychiatric and neurodegenerative disorders is substantial (104, 105). The following sections will discuss the role iPSC models have played in furthering our understanding of genetic risk variants in neuroimmunity. Please refer to Tables 2, 3 for a comprehensive overview.

**Table 2:** iPSC modeling genetic risk factors affecting neuroimmunity in neurodegenerative diseases.

Gene/genomic region and associated clinical phenotypes	Cellular function assessed	Effect of mutation in iPSC model	References
Alzheimer’s disease			
APOE: APOE4 increases risk for AD compared to APOE3; APOE2 is protective	Phagocytosis	Compared to APOE3 cells, microglia and astrocytes carrying APOE4 have reduced phagocytosis of Aβ42.	(106, 111)
	Cellular metabolism	Compared to APOE3 cells, microglia and astrocytes carrying APOE4 have more lipid droplets, increased intracellular cholesterol content and reduced uptake of LDL. APOE4 microglia also have reduced oxidative and glycolytic metabolism.	(106–110)
	Cytokine production	Compared to APOE3 cells, microglia and astrocytes carrying APOE4 secrete higher levels of pro-inflammatory cytokines in response to stimulation. APOE knockout or APOE2 astrocytes secrete less cytokines.	(106, 108, 110, 111)
	Neuronal support	APOE4 microglia reduce calcium flux and neuronal network activity in co-culture models.	(109)
TREM2: Homozygous variants associated with Nasu-Hakola disease; heterozygous variants linked to AD	Morphology	TREM2 KO microglia have less complex branching.	(112–114)
	Chemotaxis	TREM2 KO microglia have increased motility in response to ADP compared to WT cells. KO or inhibition of TREM2 also leads to reduced migration to Aβ or AD neurons and less clustering around Aβ plaques.	(114–116)
	Phagocytosis	TREM2 risk variant microglia have comparable phagocytosis of *E. coli* particles but reduced phagocytosis of dying cells, Aβ fibrils, myelin, synaptosomes. Neuronal debris accumulates in TREM2 KO microglia.	(112, 113, 116–119)
	Cellular metabolism	Microglia with TREM2 KO or TREM2 risk variants show lipid accumulation, reduced clearance of cholesterol, and lower glucose and oxygen metabolism. Other studies showed lower numbers of lipid droplets *in vitro* and reduced lipid accumulation in TREM2 variant microglia after transplantation into mouse brain.	(113, 116, 119–121)
	Calcium flux	TREM2 KO microglia have increased and more sustained calcium flux and more pronounced depletion of ER calcium stores in response to ADP, ATP, or UTP stimulation. They also have higher expression of ADP receptors P2Y12 and P2Y13. However, they show reduced calcium transients after stimulation with CXCL12.	(112, 114, 116)
	Immune activation	TREM2 risk variant microglia have reduced expression of antigen presentation and immune activation molecules.	(112, 120)
	Cytokine production	Different studies show that microglia with TREM2 risk variants or KO have comparable, reduced or increased secretion of cytokines after stimulation with LPS, LPS/ATP, LPS/IFN-γ, apoptotic cells, zymosan, or myelin.	(113, 117–119)
	Viability	TREM2 KO or risk variant microglia show reduced survival.	(112, 113, 118)
PLCG2: variants associated with reduced risk for AD		PLCG2 KO microglia recapitulate many features of TREM2 KO microglia including altered lipid metabolism, reduced cell survival, reduced phagocytosis of myelin, and reduced pro-inflammatory cytokine secretion.	(113)
SORL1: risk variants associated with AD		Microglia with SORL1 risk variants have reduced uptake of Aβ and cluster less around Aβ plaques when transplanted into mouse brain.	(122)
PSEN1: mutations linked with rare, early onset familial AD		Microglia with PSEN risk variants have increased chemotaxis and reduced cytokine secretion in response to LPS/IFN-γ stimulation.	(110)
APP: mutations linked with rare, early onset familial AD		Microglia with APP risk variants have reduced calcium transient responses to ADP. They also show increased chemotaxis and reduced cytokine secretion in response to LPS/IFN-γ stimulation. NPCs with APP risk variants accumulate Aβ40 and pro-inflammatory cytokines in long-term *in vitro* cultures. In the presence of microglia, APP risk variant interneurons produce more C3 than control neurons.	(110, 123, 124)
Trisomy of chromosome 21 (Down Syndrome) increases the risk for early onset AD		Microglia derived from patients with Down Syndrome show increased synapse pruning and impair neural network activity when transplanted into brain organoids or mice.	(125)
Amyotrophic lateral sclerosis			
C9orf72: hexanucleotide repeat expansion increases risk for ALS	Phagocytosis	C9orf72 mutant or KO microglia display reduced phagocytosis and autophagy.	(126)
	Immune activation	In C9orf72 mutant microglia, NLRP3 and nuclear NF-κB are retained longer following LPS stimulation. Transcriptomic analysis also supports increased inflammation.	(126, 127)
	Cytokine production	C9orf72 mutant or KO microglia have increased cytokine secretion in response to TLR stimulation.	(126)
	Neuronal support	C9orf72 mutation in microglia leads to significant neuronal death in co-culture model with motor neurons.	(126, 127)
FUS: risk variants associated with ALS	Immune activation	Transcriptomic analysis suggests increased inflammation in FUS variant microglia.	(127, 128)
	Cytokine production	Limited data suggests cytokine production may be altered in FUS variant microglia but not astrocytes.	(129, 130)
	Calcium flux	FUS mutant microglia show higher calcium flux in response to UDP.	(128)

**Table 3:** Patient derived stem cell models of neuro-immunity in psychiatric disorders.

Cell type	Effect of patient genetic background in iPSC model	References
Schizophrenia		
Microglia	SCZ patient derived microglia have increased phagocytosis, cytokine production, expression of drivers of inflammation and a transcriptomic signature consistent with immune activation.	(131, 132)
Astrocytes	SCZ patient derived astrocytes have increased cytokine production and a transcriptomic signature consistent with immune activation including increased antigen presentation. Compared to control astrocytes, they induce less migration of regulatory T cells.	(133, 134)
Neurons	SCZ patient derived cortical interneurons have persistent reduction in respiratory capacity after exposure to microglia conditioned media and display lower SYN1 density. Co-culture of SCZ patient derived microglia with neurons reduces neuronal spine density. If the neurons are derived from SCZ patients, synapse pruning is also increased. SCZ patient derived neurons secrete high levels of ICAM1 and have high expression of antigen presentation molecules.	(131, 132, 135–137)
Bipolar disorder		
Astrocytes	BD patient derived astrocytes have higher IL-6 production at baseline and following stimulation with IL-1β. They reduce neuronal network activity in an IL-6 dependent manner in a co-culture model.	(138)
Brain organoids	BD patient derived organoids are smaller, have reduced respiratory activity and upregulation of immune response genes including interferon signaling and antigen processing pathways.	(139, 140)
Autism spectrum disorder		
Microglia	ASD patient derived microglia transplanted into mouse brains show higher soma size and thicker primary processes.	(141)

### 4.1. Understanding monogenetic risk factors for neuropsychiatric disorders

Many neurodegenerative and psychiatric disorders have complex underlying etiology. However, certain single gene mutations are known to significantly increase the risk for certain disorders. For example, while most cases of AD are sporadic, familial cases of AD are typically caused by mutations in APP, PSEN1, or PSEN2. Additionally, GWAS have identified allelic variation in APOE or SNPs in TREM2 as strong genetic risk factors for AD (142).

#### 4.1.1. Alzheimer’s disease

Apolipoprotein E (APOE) is a lipid transporter involved in cholesterol metabolism. Its allele ε4 (APOE4) is a significant risk factor for sporadic AD while APOE2 is considered protective in comparison to the common APOE3 variant (142). Therefore, several studies have used CRISPR-Cas technology to generate iPSC with isogenic pairs of APOE2, APOE3, and APOE4 variants. These studies consistently demonstrated that compared to APOE3, APOE4 carrying microglia and astrocytes had disrupted lipid metabolism with higher number of lipid droplets, increased intracellular cholesterol content and reduced uptake of LDL (106–109). Additionally, APOE4 carrying iMG displayed reduced oxygen consumption and glycolytic activity (110). Importantly, the impact of APOE allelic variation may be more subtle in neurons and brain microvascular endothelial cells (108), highlighting the importance of glial cell dysfunction in AD. Indeed, unbiased transcriptomic and proteomic analyses have revealed significant differences in immune response pathways between APOE3 and APOE4 glia. Specifically, inflammatory signatures are already elevated at baseline in APOE4 carrying astrocytes or microglia and treatment with pro-inflammatory stimuli induces excessive pro-inflammatory cytokine secretion in APOE4 compared to APOE3 glia, while APOE2 or APOE KO astrocytes have reduced cytokine-responses (106, 111, 108, 110). APOE allelic variation has also been shown to impair neuronal support functions of glia. Direct co-culture or addition of microglia conditioned medium onto APOE3 neuronal cultures led to reduced calcium flux and impaired neuronal network activity when the microglia harbored the APOE4 variant compared to the APOE3 genotype (109). Importantly, APOE4 glia also showed reduced phagocytic uptake of Aβ42 (106, 111). Using iPSC from a patient afflicted by spontaneous AD carrying the APOE4 risk variant, Lin et al. (111) demonstrated that editing to APOE3 genotype was sufficient to improve microglial Aβ42 uptake.

Homozygous TREM2 variants are associated with Nasu-Hakola Disease—a form of early-onset dementia—while heterozygous variants are linked to AD (142). Like APOE, TREM2 is involved in lipid metabolism and a particular role for TREM2 has been ascribed to glia. iPSC-derived microglia carrying AD-associated TREM2 mutations generally display TREM2 mis-localization as well as reduced levels of mature and secreted TREM2 (117, 118, 120). Interestingly, TREM2 and APOE functions seem to be tightly interlinked. TREM2 KO iMG showed reduced expression of APOE and multi-omic analyses identified APOE as a central regulatory node affected in TREM2 KO iMG (112, 122). Hence, it may not be surprising that TREM2 variant or TREM2 KO microglia mirror several aspects of APOE variant glia phenotypes, including altered lipid metabolism and reduced phagocytosis. Microglia carrying TREM2 risk variants or TREM2 KO demonstrated impaired cholesterol clearing (113), aberrant calcium signaling (112, 114, 116) as well as lower glycolytic and respiratory capacity (116, 119). TREM2 KO iMG are also more susceptible to cell death and exhibit less complex branching than WT iMG (112–114, 118). Microglia with TREM2 KO or TREM2 risk variants consistently display reduced phagocytosis of disease-relevant particles such as Aβ fibrils, dying cells, myelin or synaptosomes (112, 113, 118, 119). Furthermore, microglial migration toward and clearance of amyloid plaques was impaired *in vitro* or when transplanted into AD mouse models (112). Interestingly, some studies found no difference in phagocytosis of *E.coli* particles (117, 118), highlighting the importance of tailoring experimental designs to the disease context. Difference in experimental setups may also explain the conflicting data on the role of TREM2 in cytokine production which has been shown to be inhibitory or activating in different studies (113, 118). Lastly, these models of AD neuroimmunity have served as a tool to identify potential therapeutic targets and validate candidate drugs in a clinically relevant model. For example, van Lengerich et al. (143) generated a modified anti-TREM2 antibody and undertook extensive characterization in mouse models to demonstrate its ability to cross the BBB and enhance TREM2 signaling. Importantly, key findings were validated using human iPSC-derived microglia suggesting its potential for pharmacological use in human AD patients.

Trisomy 21 (also known as Down Syndrome; DS) is another major genetic risk factor for the development of AD. In DS patients, Aβ deposition and tau pathology can appear in their 30 and 40s, significantly earlier than in sporadic AD cases (144). Jin et al. (125) developed an immune competent brain organoid model of DS by combining healthy control NPCs with primitive macrophage precursors from DS patients or controls. Comparable numbers of microglia were observed after 4 weeks of co-culture. Higher numbers of PSD95+ puncta in the DS microglia containing organoids indicated excessive synapse pruning by DS microglia that was also apparent when DS or control microglia were transplanted into mouse brains. Additionally, DS microglia impaired neuronal network activity which could be partially restored by blocking interferon signaling. Altered neuro-immunity has also been observed in iPSC models examining other genetic risk factors for AD such as mutations in PLCG2, SORL1, PSEN1, or APP (110, 113, 122, 145).

#### 4.1.2. Amyotrophic lateral sclerosis

Amyotrophic lateral sclerosis (ALS) is characterized by progressive loss of motor neuron function. A significant proportion of patients also display symptoms of frontotemporal dementia (FTD) (146). Several studies have employed iPSC to illuminate the role of ALS/FTD associated risk variants in neuro-immune crosstalk. Hexanucleotide repeat expansion in the intronic region of C9orf72 is the highest genetic risk factor for ALS/FTD. Comparing iMG derived from ALS patients with the C9orf72 mutation to their isogenic (reverted to normal C9orf72) counterparts, Banerjee et al. observed that mutant C9orf72 led to reduced C9orf72 protein expression (126). Indeed, C9orf72 mutant iMG were comparable to C9orf72 KO iMG in many aspects: compared to control iMG, pro-inflammatory cytokine secretion was elevated in response to TLR stimulation while phagocytosis and autophagy were reduced. Independent studies also suggested that microglia derived from ALS-patients or carrying ALS-associated mutations in FUS, are more pro-inflammatory than controls (129, 130). Importantly, this *in vitro* phenotype may contribute to the motor neuron death observed in affected patients as the C9orf72 mutation in microglia led to significant neuronal death in a co-culture model of iMG and motor neurons (126). Lastly, a recent cross-species meta-analysis found that astrocytes in ALS animal models as well as astrocytes derived from ALS patients or engineered to have ALS-associated mutations in VCP, FUS, SOD1, or C9orf72 shared transcriptomic signatures of increased inflammation and reduced neuronal support functions (127).

#### 4.1.3. Rare monogenetic disorders

Induced pluripotent stem cell models have also been incredibly successful in providing insight into rare monogenetic disorders. The following paragraph will highlight a few of these studies. Pluvinage et al. generated iPSC-derived microglia carrying NPC1 mutations that are known to cause Niemann-Pick type C disease—a rare lysosomal storage disorder affecting multiple organs including the CNS with symptoms comprising ataxia, dementia, and seizures (147). Compared to WT microglia, NCP1 mutant cells had limited lysosome formation and lipid droplet accumulation (148). By combining cell line, animal and iPSC models with postmortem human brain data, the authors were able to identify anti-CD22 antibody treatment as a potential therapeutic avenue for Niemann-Pick Disease as it was able to restore lysosome and cholesterol metabolism in NCP1 mutant microglia without affecting functionality of human iPSC-derived oligodendrocytes.

Giordano et al. assessed the impact of TREX1 or RNASEH2B loss on astrocyte function. Mutation in either of these two genes leads to Aicardi-Goutières syndrome (AGS) an inflammatory disorder characterized by increased type I interferon activity leading to neurodevelopmental delays (149). Compared to WT astrocytes, TREX1 or RNASEH2B KO cells show signs of excessive inflammation, with activation of STING and NLRP3 related pathways and increased secretion of pro-inflammatory cytokines such as CXCL8. These heightened interferon responses were likely induced by excessive DNA damage in the KO astrocytes. This phenotype is also mirrored in iPSC-derived astrocytes from AGS patients. Importantly, interferon pathway upregulation seems to be cell type specific as HC and AGS neurons are largely comparable, at least on the transcriptomic level. Supporting a disease-causing role for astrocytes in AGS, conditioned medium from TREX1/RNASEH2B KO or AGS patient astrocytes induces DNA damage and cell death in neuronal cultures. This could be prevented by using neutralizing antibodies against TNF-α, IL-1β, or CXCL8, suggesting potential therapeutic avenues.

Allison et al. examined the role of glia in spinal muscular atrophy (SMA) which is caused by loss of function mutations in SMN1 (150). Compared to astrocytes derived from healthy controls, astrocytes generated from SMA patient iPSCs express higher levels of the transcription factor GATA6 (151). Increased expression of GATA6 in turn drives upregulation of the transcription factor NF-κB as well as secretion of IL-6 and complement cascade members C1q and C3. Hyperinflammation was also observed in SMA patient-derived microglia. Importantly, SMA astrocyte conditioned medium induced neurotoxicity in iPSC derived motor neurons that could be prevented by knockdown of GATA6 in astrocytes.

### 4.2. Modeling complex genetic interplay

The above-described studies assessing the impact of single gene mutations have been tremendously helpful in furthering our understanding of disease mechanisms and identification of potential therapeutic targets. However, most patients affected by neuropsychiatric disorders do not present with a known single disease-causing genetic mutation. This is particularly true for psychiatric disorders like MDD or SCZ where genetic contribution is typically assessed using polygenic risk scores comprising several loci across the human genome. Importantly, some of the associated loci have not yet been fully characterized and the mechanistic impact on disease development is unknown. Additionally, many of the risk loci are in non-coding regions of the genome, suggesting a complex genetic interplay beyond single gene gain-of-function or loss-of-function mutations. Given that iPSCs recapitulate the complex genetic architecture of an individual, they present an ideal model system to study polygenic contributions to neuropsychiatric diseases.

#### 4.2.1. Schizophrenia

Schizophrenia is characterized by chronic peripheral low-grade inflammation and microglial activation (6–9). Indeed, several studies have shown that microglia and astrocytes derived from SCZ patients are in a hyperactive state characterized by an inflammatory transcriptomic signature, elevated secretion of pro-inflammatory cytokines and increased phagocytosis (131–133). These phenotypic alterations in microglia have important implications for neuronal function. Indeed, co-culture of neurons with SCZ derived iMG leads to significant reduction in neuronal spine density compared to co-culture with control microglia (131, 132). Importantly, studies have demonstrated that the crosstalk between neurons and microglia is impacted by the genetic SCZ background in both cell types. Indeed, a co-culture model of neurons and microglia revealed that risk variants associated with SCZ in the complement component C4 increased microglial phagocytosis of synapses when present in neurons while it did not have an effect when only present in the microglia (132). Additionally, although exposure to conditioned media from activated microglia leads to metabolic remodeling in both healthy control and SCZ derived cortical interneurons, HC neurons quickly recover while the reduced respiratory activity and capacity persists in SCZ neurons (137). Inflammatory stimulation also evokes distinct responses in SCZ iPSC derived brain organoids where treatment with TNF-α leads to a decrease in calretinin+ interneurons compared to an increase in HC organoids (152). Taken together, these data support a model where a SCZ genetic background increases sensitivity to inflammatory stimuli and simultaneously increases intrinsic inflammatory activation. Interestingly, iPSC models also suggest that SCZ specific alterations in CNS resident cells may alter recruitment of peripheral immune cells. Compared to controls, SCZ-derived neurons secreted high levels of ICAM1 (135). Elevated ICAM1 levels have been implicated in breakdown of the BBB and leukocyte infiltration to the brain and have also been associated with SCZ (153). Stimulation with IL-1β led to upregulation of inflammatory genes in both healthy and SCZ-derived astrocytes. Ensuing chemokine secretion also induced migration of regulatory T cells in an *in vitro* model (134). The migration of these generally anti-inflammatory cells was dampened when the astrocytes were generated from patients with SCZ, potentially due to limited secretion of chemokine CCL20 compared to healthy control cells. Lastly, SCZ organoids and glia were found to have transcriptomic upregulation of antigen presentation pathways (133, 136), which are important for activation of adaptive immune cells (154). Together, these data suggest that influx and activation of immune cells into the CNS may be generally increased in SCZ while recruitment of anti-inflammatory immune cells may be limited, skewing the balance towards an inflammatory milieu.

#### 4.2.2. Bipolar disorder

Bipolar disorder (BD) is characterized by periods of depression and mania (155). Peripheral and CNS inflammation is present in BD and autoimmune diseases such as Systemic Lupus Erythematosus increase the risk for developing BD (156). Many different aspects of immune signaling are altered in BD, including elevated inflammasome activity, increased indoleamine 2,3-dioxygenase levels in turn promoting conversion of tryptophan to kynurenine and high levels of reactive oxygen species to name just a few (157). Several studies have observed upregulation of immune response genes in NPCs, brain organoids, or astrocytes derived from BD patients compared to controls (138–140). For example, NPCs from BD patients have higher expression of NLRP2 (158) and BD derived astrocytes produce more IL-6 at baseline and after exposure to a pro-inflammatory stimulus (138). Co-culture models further demonstrated that BD astrocytes interrupted homeostatic neuronal network activity, which was at least partially driven by high levels of secreted IL-6. Interestingly, BD patients also have higher plasma levels of IL-6, supporting the clinical relevance of the iPSC model (7). Following this line of thought, iPSC models have also been instructive in assessing mechanisms of clinically used pharmaceuticals. Treatment of brain organoids from BD patients with lithium – a commonly used compound in maintenance treatment of BD which is difficult to appropriately study in animal models (159) – led to increased organoid size and partial normalization of electrophysiological properties that had been impaired compared to control organoids (140). A detrimental role for IL-6 has also been found in iPSC models of ASD. As in BD, ASD-derived astrocytes secreted high levels of IL-6 compared to control cells and blockage of IL-6 partially restored the detrimental effects of ASD astrocytes on neuronal synaptogenesis (160).

#### 4.2.3. Other neuropsychiatric disorders

Although ASD and MDD are strongly associated with maternal immune activation and peripheral inflammation (91, 161), to date surprisingly few studies have employed iPSC technology to assess neuro-immune crosstalk in these disorders (162). A recent study developed a xenotransplantation model of immunocompetent brain organoids into mice and found that microglia derived from ASD patients had larger soma and thicker primary processes compared to control microglia (141). Whether and how this will impact disease relevant neuronal processes remains to be determined. Additional potential exists to use iPSC-based models of neuro-immune interaction in less commonly studied disorders. For example, Tourette Syndrome, Anorexia nervosa or other eating disorders, Obsessive Compulsive Disorder or Attention Deficit/Hyperactivity Disorder may benefit from more thorough molecular studies using iPSC models.

## 5. Limitations and future potential for iPSC-based models of neuropsychiatric disorder

Despite all their advantages, iPSC-based models come with limitations. A major drawback is the loss of epigenetic information during the reprogramming process of patient biospecimens into iPSC. This is particularly relevant to models of neurodegeneration as epigenetic hallmarks of aging are erased (163). Combined with the fact that many iPSC-based models, particularly brain organoids, recapitulate the timeline of human neurogenesis and therefore largely model neurodevelopment during embryogenesis and the early postnatal period, iPSC-based models do not perfectly capture states of neurodegeneration. One strategy to overcome this limitation is to generate neurons or glia directly from other cell types without prior conversion into iPSC (164). For example, neurons derived from fibroblasts were shown to retain key epigenetic information as well as aging-associated phenotypes such DNA damage (163). Several studies have also generated microglia-like cells immediately from peripheral blood mononuclear cells, which is cheaper and technically less complex than iPSC reprogramming and iPSC culture, allowing accessibility to many different labs. Such models have for example shown that hypersensitivity of patient-derived microglia to ATP stimulation correlated with symptom severity in fibromyalgia patients (165). Importantly, this approach can also quickly generate microglia from many donors, allowing high-throughput studies of genetic variation which may be technically challenging using iPSC-based models (166). Conversely, the reprogramming of the epigenetic landscape may also be regarded as an advantage of iPSC-based models as it allows studies of homogenous cell populations without confounding variables. Genetic and environmental contributions to cellular function can be assessed in a controlled environment. It should be noted that after differentiation, iPSC-derived cell types typically recapitulate the epigenetic landscape of their *in vivo* counterpart. For example, the chromatin structure of iPSC-derived neurons strongly resembles the chromatin structure of fetal and adult human neurons, while iPSC display distinct signatures (167). Given the significant role of epigenetic modifications in neuropsychiatric disorders (168), several recent studies have examined the epigenetic landscape in iPSC-derived neuronal and glial cell types. Results highlighted significant differences in histone acetylation, DNA methylation and 5-hydroxymethylcytosine profiles comparing cells derived from healthy controls or those with neurodegenerative or psychiatric disorders (169–171). Additionally, iPSC-derived microglia models have been used to study AD-associated variants located in cis-regulatory elements (172). Undoubtedly, similar approaches will be employed more frequently in the future to study epigenetic regulation of neuro-immune interactions in health and disease.

Due to the cost and labor associated with iPSC generation and maintenance, many studies rely on very low sample numbers (e.g., iPSC derived from less than three patients) or do not include matched healthy control samples (173). While even studies with few biological replicates have yielded important insight into disease mechanisms, it is imperative that future research studies are adequately powered as interindividual variability might otherwise mask biologically relevant differences (108). This is particularly important for diseases with complex genetic architecture and unknown etiology where the patient-to-patient variation is expected to be considerable. Additionally, iPSC are usually derived from a single cell clone and hence do not reflect the genetic mosaicism in the brain. As pathogenic somatic mutations have been associated with neuropsychiatric and neurodegenerative disorders (174), future studies modeling genetic mosaicism should be of interest. Recent studies developed innovative approaches to increase genetic diversity and thereby statistical power in iPSC-based CNS models by pooling iPSC lines from various donors in one dish. Combined with single cell RNA sequencing, identification of eQTLs is possible (175–177).

The coming years will see technical advancements in modeling neuroimmunity. For example, novel protocols are employing three-dimensional microfluidic devices to better mimic neuro-immune interactions (178), integrate vasculature like structures into brain organoids (179) or develop models of traumatic brain injury in brain organoids (180). Future studies will certainly also increase the number of cell types and states that can be modeled using stem cells. Indeed, current iMG models do not reflect the spatial heterogeneity of microglia in the brain and protocols for generating non-microglia CNS resident myeloid cells such as CNS-macrophages do not exist despite their distinct roles in brain homeostasis and disease (181, 182). When transplanted into mouse brains, human iPSC-derived hematopoietic progenitor cells migrated to various sites in the brain and acquired niche-specific phenotypes including characteristics of meningeal, perivascular, or choroid plexus macrophages (43). Future studies employing similar approaches and the development of protocols to generate CNS-macrophages directly from iPSC will be essential in furthering our understanding of these cell types in human CNS disorders.

While most iPSC-based models of neuro-immune crosstalk have so far focused on key neurodegenerative (AD, ALS) and psychiatric disorders (SCZ, BD), the use of patient-derived stem cells will certainly be expanded to interrogate the interplay of genetics and neuroimmunity in a broader range of diseases. Key areas of interest include other neurodevelopmental and psychiatric disorders such as MDD or ASD, particularly since inflammation has been implicated in disease etiology (91, 161). The upcoming years will likely also see innovative solutions to model other environmental risk factors with iPSC such as the exposure to heavy metals or pollution (Figure 2). Models to investigate the gut-brain-immune axis in psychiatric disorders will also be of interest (183, 184).

Lastly, stem cell models hold great potential to identify disease-relevant therapeutics targeting the neuro-immune axis. High-throughput screening platforms have already been employed in iPSC-derived neuronal cell types, for example to evaluate drugs suitable for repurposing as antivirals against ZIKV (84). In the case of neurodegenerative disorders, several drugs identified and tested in iPSC-based neuron and astrocyte models have now advanced to clinical trials (164, 185). Similar approaches will be useful to identify microglia targeting therapeutics.

## Author contributions

CM: Writing – original draft. ZW: Writing – review & editing.
